# GaitKeeper: A System for Measuring Canine Gait

**DOI:** 10.3390/s17020309

**Published:** 2017-02-08

**Authors:** Cassim Ladha, Jack O’Sullivan, Zoe Belshaw, Lucy Asher

**Affiliations:** 1 Centre for Behaviour and Evolution, Henry Wellcome Building, Newcastle University, Newcastle NE2 4HH, UK; J.O’Sullivan4@newcastle.ac.uk (J.O.); lucy.asher@ncl.ac.uk (L.A.); 2 School of Veterinary Medicine and Science, University of Nottingham, Sutton Bonington Campus, Leicestershire LE12 5RD, UK; z.belshaw.97@cantab.net

**Keywords:** gait, locomotion, stride segmentation, accelerometer, dog, exercise, movement science, inertial measurement unit (IMU), gyroscope

## Abstract

It is understood gait has the potential to be used as a window into neurodegenerative disorders, identify markers of subclinical pathology, inform diagnostic algorithms of disease progression and measure the efficacy of interventions. Dogs’ gaits are frequently assessed in a veterinary setting to detect signs of lameness. Despite this, a reliable, affordable and objective method to assess lameness in dogs is lacking. Most described canine lameness assessments are subjective, unvalidated and at high risk of bias. This means reliable, early detection of canine gait abnormalities is challenging, which may have detrimental implications for dogs’ welfare. In this paper, we draw from approaches and technologies used in human movement science and describe a system for objectively measuring temporal gait characteristics in dogs (step-time, swing-time, stance-time). Asymmetries and variabilities in these characteristics are of known clinical significance when assessing lameness but presently may only be assessed on coarse scales or under highly instrumented environments. The system consists an inertial measurement unit, containing a 3-axis accelerometer and gyroscope coupled with a standardized walking course. The measurement unit is attached to each leg of the dog under assessment before it is walked around the course. The data by the measurement unit is then processed to identify steps and subsequently, micro-gait characteristics. This method has been tested on a cohort of 19 healthy dogs of various breeds ranging in height from 34.2 cm to 84.9 cm. We report the system as capable of making precise step delineations with detections of initial and final contact times of foot-to-floor to a mean precision of 0.011 s and 0.048 s, respectively. Results are based on analysis of 12,678 foot falls and we report a sensitivity, positive predictive value and F-score of 0.81, 0.83 and 0.82 respectively. To investigate the effect of gait on system performance, the approach was tested in both walking and trotting with no significant performance deviation with 7249 steps reported for a walking gait and 4977 for a trotting gait. The number of steps reported for each leg were approximately equal and this consistency was true in both walking and trotting gaits. In the walking gait 1965, 1790, 1726 and 1768 steps were reported for the front left, front right, hind left and hind right legs respectively. 1361, 1250, 1176 and 1190 steps were reported for each of the four legs in the trotting gait. The proposed system is a pragmatic and precise solution for obtaining objective measurements of canine gait. With further development, it promises potential for a wide range of applications in both research and clinical practice.

## 1. Introduction

Detailed analysis of the gait cycle in humans has demonstrated its utility as a window into many underlying neurodegenerative and medical diseases, as well as biomechanical conditions [1,2,3]. The translation of such methodologies to non-human species can be problematic due to the need to account for their different physiological and cognitive abilities.

Parameters (or micro-gait features) in the human gait cycle that have been identified as sensitive predictors of disease (in a medical sense) can be roughly characterized into spatial or temporal categories: temporal parameters pertaining to intra-leg based timing events (e.g., velocity, length, width), and spatial parameters being related to measurements between the environment and skeletal features (e.g., pitch, roll, joint flexions) [4,5]. In either case, the calculation of each micro-gait feature starts with the precise detection of final and initial contact times (FC and IC, respectively) of the foot with the floor. These events can be directly used to determine when the foot is in stance (between IC-FC) or swing (FC-IC) and thus form the basis of other more complex calculations to determine both temporal and spatial micro-gait features in addition to gait asymmetry (difference inter-leg) and variabilities (difference intra-leg) [6,7,8]. In humans, these parameters have been shown to correlate with neurological and physiological conditions [1,9].

In recent years there has been an increase in interest in the application of similar measures of gait for use with companion animals. In the United Kingdom approximately 30% of households own a dog (Canis familiaris) [10]. Many of these animals either currently suffer from or are at risk of developing orthopaedic diseases; it is estimated 20% of dogs over 1 year old have osteoarthritis [11]. The potential impact of a tool for the prediction and diagnosis of such conditions, and the monitoring of recovery post-treatment, could be substantial. Currently the most prevalent form of gait analysis performed by veterinarians are forms of subjective visual gait assessment [12]. Numerical rating scales and visual analogue scales are two subjective, observation based methods which are used to evaluate the severity of lameness [13]. A wide range of clinical scales have been described [14] to assist the grading of canine lameness in clinical veterinary practice but few are validated, therefore their reliability and value is unknown. These two subjective measures have been shown, despite their ubiquity, to lack accuracy in the majority of instances except where lameness is severe [12,15].

Technologies for automatically measuring gait, in both humans and dogs, can be placed in two categories: worn and un-worn [16]. In the un-worn category are methods ranging from manual coding of videos to the use of force plates and camera based 3D motion analysis. Force plates are often used in research centres or specialist veterinary referral clinics to measure the ground reaction forces which are generated when dogs stand on or walk across the measurement region [15,17]. They are generally termed kinetic-based measurements and are currently considered the gold standard in animal movement science as they provide an objective and precise method of collecting foot-down, gait related measurements [7]. Unfortunately, while precise, this category of movement detection is limited to instrumented environments, which may be stressful for the dog, un-natural and dimensionally constrained [18]. In addition, the specialist nature of the equipment often means limited, expensive availability in sparse geographic locations.

Worn sensors are widely researched in human movement science with focus on improving existing measures [19], developing new ones such as foot clearance [20] and characterising conditions by gait [21,22]. In human movement science, the objective outputs available from worn sensors are approaching a maturity whereby they are capable of informing clinical decision making process for movement related conditions [23]. These sensors measure the movement of the supporting limb or body rather than forces and are generally termed kinematic measurements. While some research has been done using flex sensors and/or pressure transducers [24,25], the most common worn sensor systems combine accelerometers and gyroscopes; this combination is termed an inertial measurement unit (IMU). The popularity of the device in the clinical setting, both among humans and animals, has been accompanied by the rise in commercial products for humans (e.g., FitBit), or tailored alternatives for dogs (e.g., Whistle) [26,27]. Unfortunately, these devices, although low cost, employ black box methodologies obfuscating their strengths and weaknesses which therefore prevent the in-depth analysis of their potential for application in a research or clinical setting [26,27,28]. More recently, the research community has recognised these shortcomings and has begun to embrace open-source, transparent capture systems [29].

In human movement science, it is common for patients to wear an IMU and to perform a structured movement protocol laid out by the clinician. Protocols are varied but a very common one is the continuous walk test (CWT) [30] which is designed to assess the physical capacity of the subject. Variants of the test (such as asking the patient to count while walking) are also common and designed to stress neurological pathways; accentuating disturbances in the gait [31]. As the tests are common practice and standardized, bench-marks exist where bounds for age-related norms are in place. This makes clinical measurements easy and un-complex.

In animal movement science, researchers have experimented with IMUs since they became ubiquitous during the 2000 s [32], however, to date the widest adopters of them in clinical practice is the equine industry. These equine systems are presently being used in clinical practice for lameness detection and interoperability between systems is reported to be improving [33]. Of the equine-based systems in use, there seem to be no standardised protocols (equivalent to the CWT) and subsequently a lack of openly available and reliable bench marking (although some work has been done with hoof-mounted impact sensors [34]). To the authors’ knowledge, no systems are available for measurements in dogs.

It is clear that IMU-based gait measurement could result in valuable insights to normal and pathological animal movements. The relatively low cost and ability to deploy worn sensors over both short and extended periods of time could make IMU gait measurement an enabling technology to detect and monitor gait abnormalities in dogs, in companion animals [35,36,37]. If this led to affected animals receiving appropriate veterinary treatment at an earlier stage, this could have a significant positive impact on their welfare.

Initial studies have demonstrated IMUs’ benefit as a clinical tool in equine science [38,39,40,41,42]. However, to make such measurements necessitates precise and well-characterized instruments that are validated in the intended use environment.

This paper describes the signal-processing approach used to detect final and initial contact times of dog paws. Particular attention is paid to the tailoring the development to deal with the idiosyncrasies of the canine gait cycle. Results are given from an experiment involving the comparison of the system’s step detection to the manually coded steps identified within high-speed video by two trained observers. Both step prediction and step annotation were performed on the same walking bouts for each dog allowing for a one to one comparison. The cohort used consisted of 19 dogs of a variety of breeds. Performance of the step detection methods were calculated as the Positive Predictive Value, Sensitivity and the F-measure. Potential sources of error within dogs were investigated through the use of a mixed linear model. The system was evaluated independently for both walking and trotting gaits.

## 2. Materials and Methods

The experimental design described was approved by the Newcastle University ethics committee. Data analysis was performed using custom Matlab scripts developed by Cassim Ladha.

### 2.1. Data Collection

A convenience sample of 19 healthy adult dogs was recruited from the local area using posters and local advertising. The sample contained breeds and crossbreeds of Border Collie, Cavalier King Charles Spaniel, Cocker Spaniel, Corgi, Doberman, Irish Wolfhound, Jack Russel Terrier, Labrador, Lurcher (with various mixes of breed), Poodle, Rhodesian Ridgeback, and Springer Spaniel. All dogs were deemed not to have any major gait impediment though retrospective video review by a qualified veterinary general practitioner.

Experiments took place in a disused University residence hall. A 15 m long linear track was marked out using tape on a hard, non-slip parquet wooden surface. Before data capture commenced, each dog was given the opportunity to familiarise itself with the room used for experiments. The dog was judged to be familiar with the surroundings and ready to proceed when investigation of the environment had ceased and their demeanor was judged to be both calm and comfortable by the researchers. The height of the dogs, measured in cm from the floor to the withers, was taken at this point. The owners also provided details as to the age of their dog(s) and with one exception (whom reported mild wrist arthritis), confirmed that the dogs had no known medical issues.

**Figure 1:**
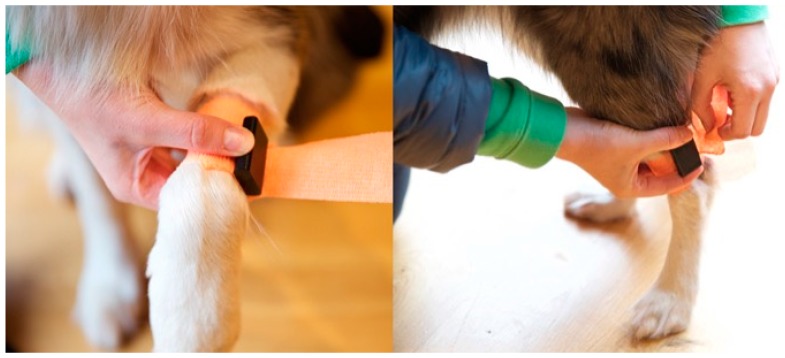
Sensors were attached in the positions shown and secured with coloured cohesive bandages.

As a control, dogs were first walked un-instrumented with a standardised lead for six laps, three clockwise and three anti-clockwise. The walk was videoed with a high-speed hand held camera at 240 frames per second (FPS); iPhone 6s (Apple Inc., Cupertino, CA, USA). Subsequently, each dog was then instrumented using four IMU sensors (Vetsens, Newcastle-upon-Tyne, UK); one on each leg. This was performed in two stages. Firstly, a cohesive bandage (Vetrap, 3M, Bracknell, UK) was wrapped twice around the prospective sensor location (above the carpal joints of the thoracic limbs and below the tarsal joints of the pelvic limbs) as shown in Figure 1. The dogs were given a short time to habituate to wearing them and once deemed they had done so, sensors were attached to the lateral limb aspect using a second layer of cohesive bandage (placed directly over the first).

**Figure 2:**
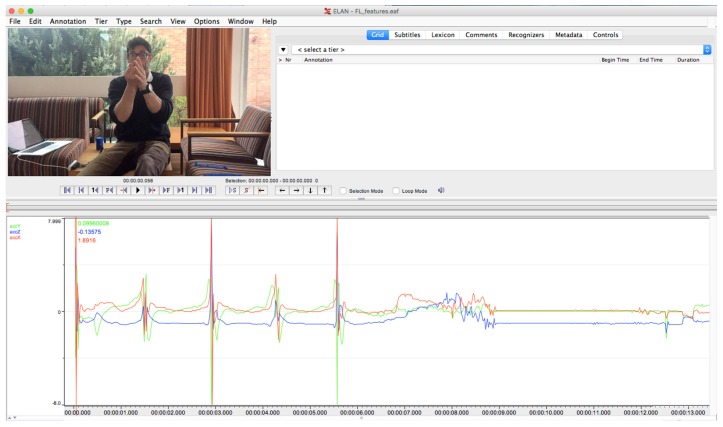
Sensors and video were synchronized on short successive taps, which appear as sharp spike in the accelerometer data stream.

Each IMU sensor contained a 3-axis gyroscope and accelerometer. Both were simultaneously sampled at 100 Hz and output wirelessly over Bluetooth to a laptop (Apple Macbook Pro, Apple Inc., Cupertino, CA, USA). Received data was aggregated into a log-file for post processing. To provide a distinct start point, within both video and IMU data the sensors were videoed being tapped five times. These taps were clearly visible in the video and could be identified in the accelerometer data as short, distinct impulses; see Figure 2.

These were repeated after the walk, prior to stopping the data and video collection. Once this had occurred the sensors were attached with a layer of cohesive bandage (over the one that previously applied). Sensors were attached in a consistent orientation; USB port facing downwards and sited on the lateral aspect of the limb. The bandages of the left and right limb pairs were coloured differently (Left: green, Right: red) to assist in their differentiation during the subsequent annotation of the high speed videos. They were judged to be fully habituated when researchers observed a return to the gait pattern displayed prior to bandage attachment. Once the researchers conducting the experiment were satisfied that initial loss of proprioception (caused due to the leg bandages) had subsided and the dogs were walking with a consistent gait, video and data capture was started. At this stage the dogs were made to repeat the same walk protocol as used when not instrumented.

### 2.2. Processing of IMU Data

**Figure 3:**
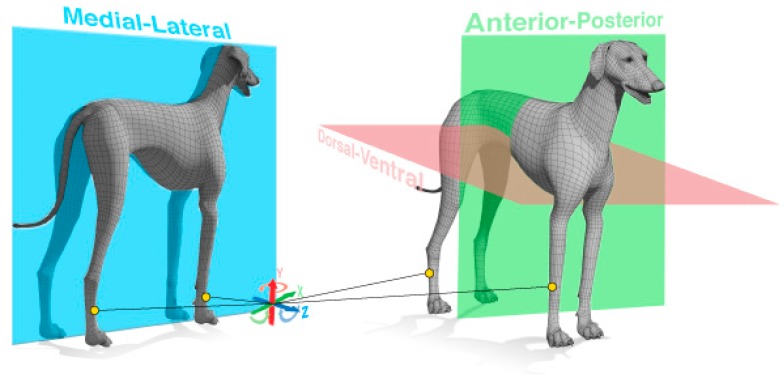
Cartesian to animal centric coordinate system (Anterior = A, Posterior = P, Dorsal = D, Ventral = V, Medial = M, Lateral = L). Direction of the arrow discerns positive axis. End mapping was +X = AP, +Y = DV, +Z = ML). Sensors were placed laterally on each leg such that the medial-lateral axis were aligned.

In a pre-processing stage before any analysis, IMU data pertaining to each leg was time-synchronized based on alignment of the taps inserted into the accelerometer signal. Next, data from each leg was re-labeled out of Cartesian space and into a dog-centric coordinate system consisting of; dorsal-ventral (DV), medial-lateral (ML), and anterior-posterior (AP) axes (see Figure 3). During mounting the sensors, careful attention was given to ensure the sensors positive z-axis was always aligned with the positive ML axis. This was achieved by mounting sensors on the left legs back-to-front. The importance of this step ensured similar signal features (troughs) could be used for both left and right legs. The aligned and reoriented data was then analyzed on a leg-by-leg basis through a number of operations that automatically labelled signal features pertaining to the point the foot left the floor (final contact, FC) and the point the foot hit the floor (initial contact, IC). In high-level terms, the analysis scripts identified FC events through spotting peak features that correspond to the foot initiating movement from the end of a stance phase. Subsequently, IC contacts are then found when the foot stops moving and comes to rest (velocity drops to zero) at the end of a swing phase.

**Figure 4:**
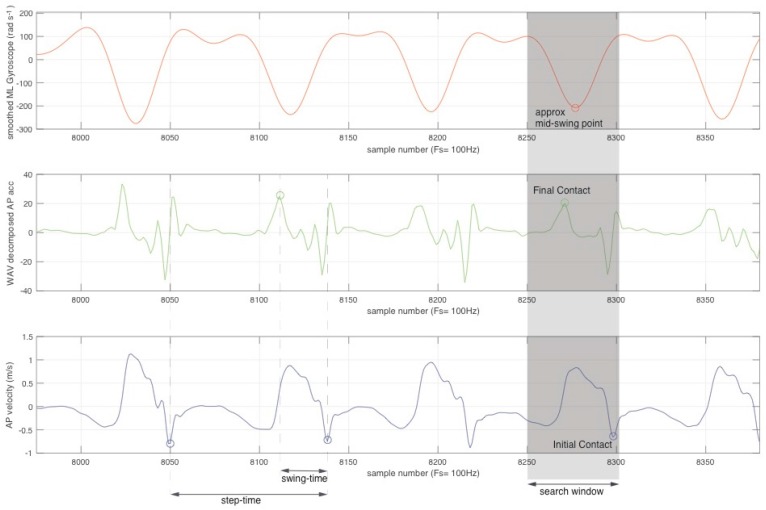
Transformed signal traces and diagrammatic representation of how FC and IC events are detected. From the FC and IC events, step-time and swing-time can be inferred.

The gyroscope data aligned in the AP axis was smoothed with a 4th order Butterworth filter (f_c_ = 5 Hz). The result of this operation is a roughly sinusoidal signal with troughs around the mid-swing point of each step. A window was placed around each trough and used to highlight possible locations where FC and IC events could occur. The search window width was derived from results reported in Ladha et al. [43]. For FC times, the AP acceleration was decomposed using a 2nd order sym4 wavelet. FC events were detected as high-points, preceding the ML gyroscope trough and within the search window. These points were manually verified using synchronized high-speed video to corresponded to the limb initiating movement from a stationary position (stance) into swing. To determine IC events the AP acceleration was integrated using a Runge-Kutta based approach to yield velocity. To cater for integration drift, the integration was reset for each step; only the signal within the search window was considered. Velocity was subsequently smoothed IC events; found as low-points preceding both the paired FC event and the ML gyroscope trough within the search window. A diagrammatic representation of the event detection process is shown in Figure 4 with example signal traces.

Once FC and IC events were identified, steps were delimited between subsequent IC events on the same leg; swing time was calculated as the period between FC and IC events and stance time as the period between IC and subsequent FC events.

### 2.3. Video Annotation

**Figure 5:**
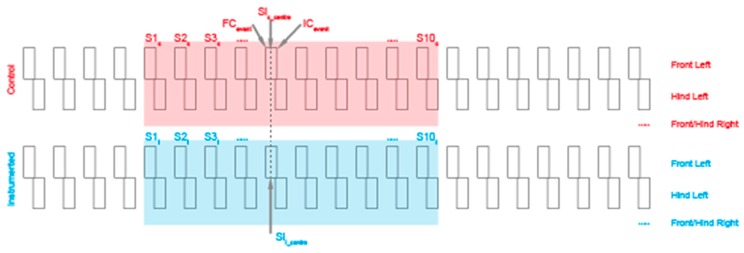
Approach for determining agreement between prediction and annotation.

All videos taken during the experiment were manually annotated by two observers using the Elan software (Max Planck Institute, Nijmegen, The Netherlands). In the annotation process, each leg was considered separately and swing phase for each step marked as per the ethogram in Appendix A. Swing events that were obscured, out of shot or unclear were ignored. The rationale behind annotating swing phase was that it is an easy feature to visually pick out; it enables extraction of FC and IC events as start and end points of the annotation respectively with little room for ambiguity. For the control walks, 11 consecutive walking strides were annotated for each dog to enable the extraction of 10 consecutive step start and end points for each leg (see Figure 5). This quantity of steps was chosen as all dogs in our test cohort (no matter of size) were able to complete without accelerating or decelerating towards the track limits. Videos of the instrumented walking bouts were annotated fully with every step included. The initial and final synchronization taps were also annotated to allow synchronization of the annotation with the data provided by the sensors. Gait type was also noted as either walk or trot as per the ethogram in Appendix A.

### 2.4. Inter-Rater Reliability

To ensure annotation quality a sub-selection of five dogs non-instrumented walks were annotated by two annotators. The annotation files were then imported into Matlab using the SALEM toolbox (Hanheide, Lohse and Dierker et al. [44]). A custom script was written to match by time each pair of swing phase annotations. The script also gave feedback as to any missing or additional steps in either annotator’s files. The midpoints of each annotated swing phase were calculated (as the time between FC and IC) and the mean time difference, in seconds, between annotators was calculated. The standard error of the mean time difference was also calculated. Provided together these metrics give an indication of both the accuracy and consistency of annotations between annotators.

### 2.5. Effect of Instrumentation

To determine if wearing the sensing equipment had any effect on normal gait pattern a comparison of control walks to instrumented walks was performed across all dogs. The lack of instrumentation during the control phase meant only the duration of gait features could be used for comparison. The instrumented and control annotation files were imported to Matlab using SALEM. The mean step duration, defined as the time between consecutive IC events, were calculated for both files for each dog. The instrumented and control mean durations were then correlated using a Pearson’s correlation to give an indication as to the amount of divergence between the two walking bouts. This was taken as an indication of the effect of the instrumentation on the gait. The same method was repeated using the mean swing durations. This was to investigate whether, not only was the step duration unaffected but, the composition of the step remained consistent with the normal gait post-instrumentation.

### 2.6. Comparing Annotations and Predictions

To assess the performance of the system, at step detection, we first compared annotated steps to detected steps. This was performed using Matlab scripts which imported the annotation file and the pre-processed step prediction data. As videos were captured and viewed in high speed the annotation files first needed adjusting to match the times provided by the sensors. The time origins of the two data sets were then aligned.

To avoid the problem of neighboring steps being misinterpreted as false positives (FP), our approach involved finding the center point between Final Contact (FC) and Initial Contact (IC) on the annotated steps. A time window the width of the maximum swing time observed within the dog was then centered on this center point. This window provided the search area for the corresponding swing phase mid-point in the predictions. The true positives (TP), FP’s and false negatives (FN) were recorded. TPs were defined here as those annotated steps with a single predicted step match. FPs were identified as those steps of either the predicted or annotated datasets with multiple matches in the other dataset and those in the predicted dataset without a corresponding annotated step. FNs were identified as those annotations that could not be matched with a corresponding prediction. TP, FP and FN counts were collected on a per-leg, per-dog basis. The three metrics were summed to receive a total value of each which encompassed the performance of the prediction algorithm across all legs and dogs. These were then used to calculate the Positive Predictive Value (PPV) and sensitivity of the algorithm:

(1) $$\mathrm{PPV}=\frac{\mathrm{TP}}{\mathrm{TP}+\mathrm{FP}}$$

The sensitivity is a metric of the proportion of steps correctly identified as steps and can be calculated as:

(2) $$\mathrm{Sensitivity}=\frac{\mathrm{TP}}{\mathrm{TP}+\mathrm{FN}}$$

The F-score gives an overall indication of (but is not synonymous with) the accuracy of the prediction method [45]. Here the traditional balanced F-score is used which is the harmonic mean of PPV and sensitivity and is calculated as:

(3) $$F-\mathrm{Score}=2\cdot \frac{\mathrm{PPV}\cdot \mathrm{Sensitivity}}{\mathrm{PPV}+\mathrm{Sensitivity}}$$

### 2.7. Performance Bias

A preliminary investigation of possible sources of performance bias was performed through the use of a Linear Mixed Effects Model. The outcome measure was chosen as the error between annotated and predicted swing phases, measured in seconds. The height to withers (HTW-linear), age (linear) and sex (two levels: male or female) were chosen as potential sources of bias in the algorithm’s performance. The subject was included as a random effect to account for any differences that were not collected and accounted for here. The leg the sensors were attached to was also included as a nested variable within the subject. The model formula was therefore:

*Error* ~ *HTW* + Sex + *Age* + (1|*Dog*/*Leg*) (4)

Four other variations of this model were constructed for comparison using likelihood ratio tests. Each of these variations removed one effect variable. Models were run and analyzed in R (Version 3.3.1, R Core Team, Vienna, Austria, 2015) using the LME4 package [46].

## 3. Results

The average time taken to make a measurement on the dog was 17 min. This was typically broken down as: 3 min to talk to the owners; 3 min to conduct the control walk; 5 min for the habituation to equipment; 1 min to set-up instruments once habituated; 2 min to attach sensors; and 3 min to conduct the instrumented walk. The processing of the data using Matlab scripts was a matter of seconds and the output was a data file that could be used for post-analysis and to generate a report.

**Figure 6:**
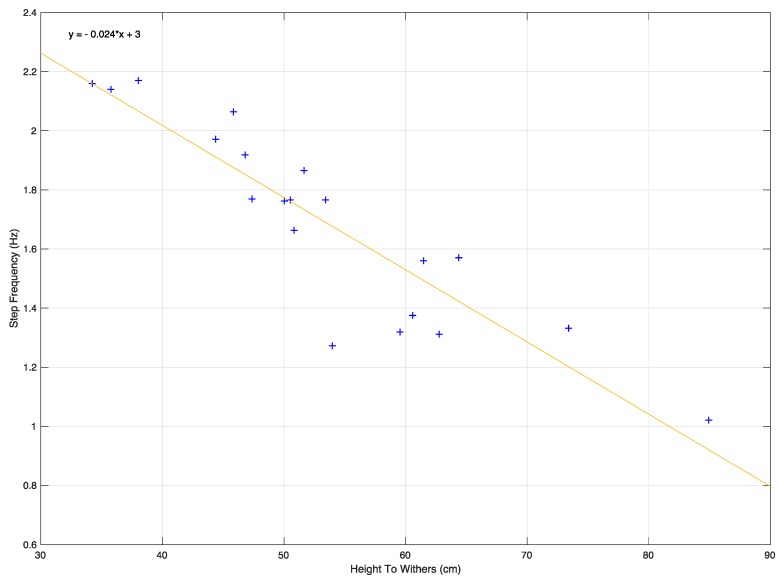
Plot of height to withers vs. step frequency. The linear relationship closely resembles the one previously reported in Heglund et al. [47].

Data from all 19 dogs was successfully captured with the sensors and subsequently analyzed. Across all four legs of all nineteen dogs, a total of 15,660 steps were annotated. Step frequencies of the four legs, across all dogs, were 3922, 3844, 3780 and 3937 for the front left (FL), front right (FR), hind left (HL) and hind right (HR) legs, respectively, and as such do not appear to differ. However, the number of steps taken did appear to vary greatly between dogs with a range of 489 steps per dog to 1255 steps per dog and a mean of 824 steps per dog (230 SD). Step frequency was significantly negatively correlated to the HTW of the dog (r = −0.903, n = 19, *p* < 0.001; Figure 6) which is consistent with Heglund et al. [47].

### 3.1. Inter-Rater Reliability

The videos captured were annotated by two trained annotators using the same ethogram (Appendix A). No mismatches between annotators were reported when identifying steps; perhaps because of the very obvious leg-swing. The mean difference in the swing phase mid-points recorded by the annotators was 0.016 s (±0.000 Standard Error of the Mean (SEM)) which corresponds to 3.8 frames at the video capture rate. This is approximately equal to 5% of the mean swing phase duration (0.315 s) which falls well below the 10% degree of error deemed acceptable. The low error and SEM values suggest the annotations made were highly similar and demonstrated a high level of consistency.

### 3.2. Effect of Instrumentation

A visible change in locomotion was noticeable immediately after the bandages were attached. No dogs in the cohort showed any signs of pain or discomfort but all showed a varying degree gait abnormality (increased step height and variable pace), although the effect was transient. All dogs seemed to habituate to the instruments within a 3–10 min time span and habituation was found to accelerate with dog mobility. For this reason, encouragement with use of toys or treats was given to move during habituation.

**Figure 7:**
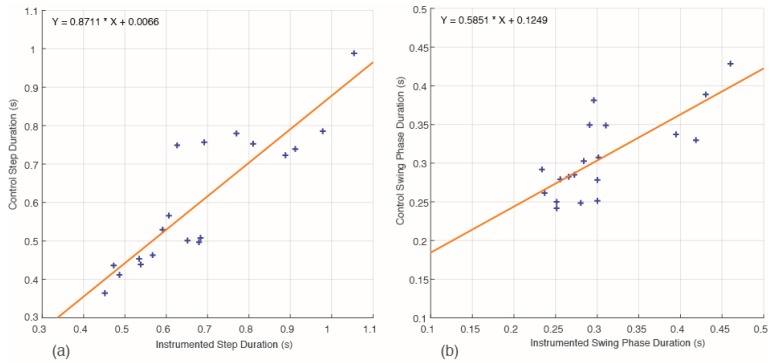
(**a**) Pearson’s correlation between Control and Instrumented step duration (r = 0.88); (**b**) Pearson’s correlation between Control and Instrumented swing duration (r = 0.76).

To quantify the effects of instrumentation the average step durations of those steps annotated for the control and instrumented walks were correlated against each other for each dog. The same was performed for the average swing phase durations. The step duration showed a strong significant correlation (r = 0.88, n = 19, *p* < 0.001; Figure 7a). The swing phase duration also gave a strong and significant correlation (r = 0.76, n = 19, *p* < 0.001; Figure 7b).

### 3.3. Comparing Annotations and Predictions

A total of 15,671 steps were predicted to have occurred across all dogs and legs. Of these a total of 12,678 were accepted as TPs (80.9%) and 2993 were rejected as FP (19.1%). 2680 of the 15,660 annotated steps were not matched with a prediction and were therefore labelled as FN (17.1%).

Of the steps accepted as TP, 7249 were annotated as walking with 1965 of these recorded from the front left (FL) leg, 1790 from the front right (FR), 1726 from the hind left (HL) and 1768 from the hind right (HR). 4977 TP steps were annotated as trotting of which 1361 were from the FL, 1250 from the FR, 1176 from the HL and 1190 from the HR.

**Figure 8:**
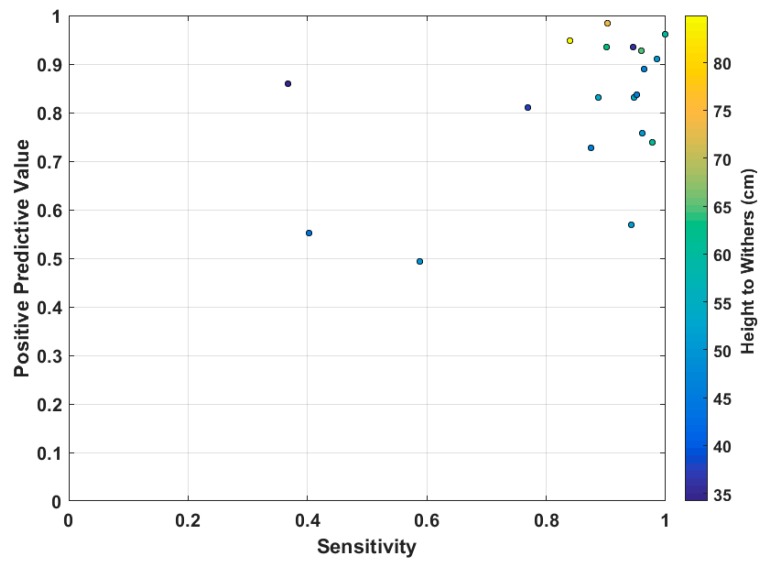
Results of PPV and Sensitivity calculations showing four outliers and no significant interaction between height and system performance.

The PPV for all dogs and legs was 0.81 and Sensitivity 0.83. The resultant F-Score was 0.82. Figure 8 shows a PPV-Sensitivity plot of each dog’s performance with each dog color coded by their HTW. As can be seen the 16 of the dogs are grouped in the top right section of the graph. There are however, three outliers which report poor Sensitivity scores. Two of the three outliers are those with the lowest HTW measurements.

The exclusion of these outliers results in an improvement in overall performance. The PPV improves by 0.03 to 0.84 and the Sensitivity improves more significantly, by 0.09, to 0.92. The F-Score also improves to 0.88.

### 3.4. Processing of IMU Data

**Figure 9:**
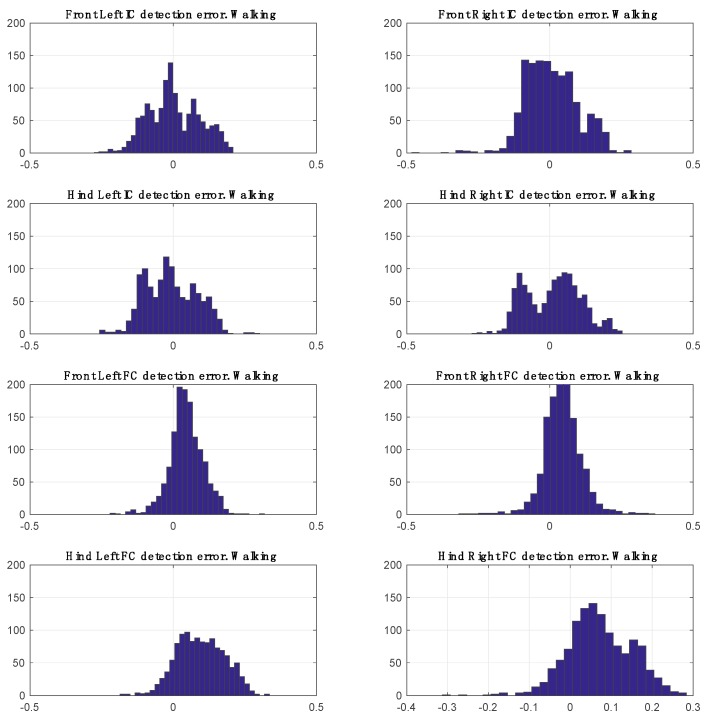
Disagreement between predictions and annotations during walking.

**Figure 10:**
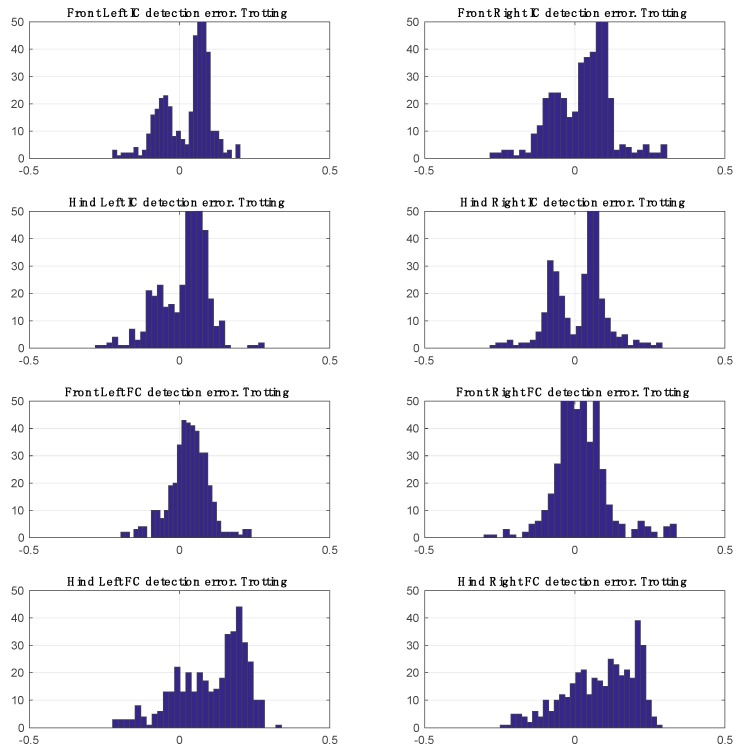
Disagreement between predictions and annotations during trotting.

The total number of steps used for performance evaluation was 5041 (FL = 1312, FR = 1310, HR = 1217, HL = 1202). Mean errors for FC and IC detection are listed in Table 1 and distribution of disagreement between annotations and predictions are shown graphically for walking and trotting in Figure 9 and Figure 10.

For both walk and trot gaits, the results demonstrate that there is no significant bias towards left or right paws. It is also clear that there is no bias between front and hind limbs for initial contact detection. The performance for final contact detection is slightly better on the fore limbs than the hind limbs.

### 3.5. Performance Bias

A total of five statistical models were built for comparison. The first was the full model as stated in the formula above. Three other models were constructed, each identical except in their omitting of one of the fixed effects (HTW, Age and Gender). The final model dealt with the effect of the Dog random variable and required the expansion of the full equation given above to the long form. Each of these were compared to the full model with LRTs.

Age was not found to have a significant effect on error (χ2(1) = 1.9991, *p* = 0.1574), neither were HTW (χ2(1) = 0.698, *p* = 0.4035) or sex (χ2(1) = 0.3496, *p* = 0.5543). However, the dog random effect was found to be highly significant (χ2(1) = 21.538, *p* < 0.001).

## 4. Discussion

This paper introduces what we believe is the first sensor based gait analysis targeted towards canines. The system shows promise to enable field based measurements conducted in non-clinical settings. This would therefore enable the combination of both clinic-based assessments and longitudinal measurements when the dog is in a home environment. Such systems have demonstrated their utility in human clinical practice [35] and to some extent with equine species [29,34]. From a usability standpoint, the system was easy to configure and after brief temporary gait alterations (after the devices were attached to the dogs’ limbs), the subjects showed good compliance. While the cohesive bandage solution did not appear to allow for sensor movement on the limb, it is considered an area where improvement towards practicality could be made. The main concern with the cohesive bandage approach is the usability it affords within a busy clinical setting; a specialized sock that constrains sensor orientation and position but doesn’t restrict movement would be more favorable. A specialized sock would also have the benefit of added standardization the position of the sensors around the circumference of the limb and with respect to the joint. Within this experiment neither were controlled for and are an anticipated source of error.

The initial step to validate the system was to provide evidence that the sensors themselves did not affect the normal gait of the subjects. The strong, significant correlations of both the entire step duration and the swing phase demonstrate that instrumentation has no significant effect on the temporal aspects of the dogs’ gait.

Results demonstrate a good capability to delineate steps and reject shuffles or stumbles (which produce signals unclassifiable by the FC/IC detection methods). In total, three signal features were used to segment out final and initial contact times for thoracic and pelvic limbs. The features were chosen for their high-contrast properties; which makes for robust automatic extraction and low opportunity for false positives. Performance, both before and after the exclusion of the outlying dogs, was promising. The F-scores provide a simple, single number impression of the prediction methods ability and indicate that the system is effective at measuring gait micro-features. To provide a more detailed impression of the performance these should again be broken down to their constituent parts. The PPVs both pre- and post-exclusion indicate the script can be said to be correctly identifying between 81% and 84% of its identified steps correctly. The sensitivity scores of 0.83 and 0.92 indicate that those steps identified correctly represent 83% and 92% of the total steps annotated. Put more simply, and in terms of the better performing model, of the steps reported as positive approximately 84% will be TP and those will directly correspond to 92% of the number of the potential total TPs. To put this context a recent paper studying the sensitivity of a force plate method in dogs to distinguish low grade lameness, found a sensitivity of 0.63 [48].

The cause of the three outliers’ poor performance scores remains ambiguous; no apparent pattern emerged between them. The linear mixed effects model excluded some potential candidate causes and furthermore, the error values presented in Table 1 suggest no bias towards the left or right, or the pelvic or thoracic limb pairs. Alternative hypotheses to the source of the outliers warrant further investigation.

Sensor and video synchronization could partially explain disagreements between annotations and predictions. The video was captured at 240 fps giving a temporal resolution of ~4ms between frames. The sensor data was captured at 100 Hz giving a temporal resolution of 10ms between samples. Synchronization between video and sensor was achieved through identifying the frame that corresponded to a tap on the sensor. However, as the sensor has three axes a tap impulse may not necessarily result in sample-aligned spike on all of them; the x axis was arbitrarily chosen for synchronization purposes. Additionally, when making taps on the sensor, no control was placed on the orientation of the sensor. This setup results in a possible uncertainty margin of 14 ms. As the sensor sample rate and video frame rate were constant, once synchronization was performed any time misalignment would remain consistent throughout that capture period and thus any synchronization based error would be systematic over all measurements. Therefore, when making measurements outside of an experimental environment, synchronization errors of this nature would not necessarily be of any issue and would not affect the usability of the system or the repeatability of results.

The experiments described were designed to assess the IMU’s suitability at precisely detecting final and initial contact times. Although not of interest in isolation they are the fundamentals of calculating many parameters [6,34] including step time, stance time, swing time. Asymmetries and variabilities in these measures have been shown in human studies to be good predictors of various disease states. Thus, a system that can objectively measure with repeated precision final and initial contact times is likely to be of interest to both the canine research and veterinary communities. In addition, based on accurate dissection of step phase, parameters such as jerk, velocity, length and width can be calculated based on other signals directly available from the IMU sensor [9,49,50,51]. Such parameters are readily available from instrumented walkways and used for assessing lameness. An equivalently precise system that can be used in an unconstrained environment thus has the potential to be of interest to veterinary gait specialists [39,52].

The approaches presented could be integrated into a system that has potential to objectively quantify gait. It is anticipated that the margins of measurement error are sufficiently low such that subtle gait abnormalities can be detected. With careful design of a data-collection protocol, measurements made over a longitudinal time frame could be compared. Presently, the methods are untested outside of a controlled environment and it is anticipated that several challenges would need to be overcome before deployment in such a scenario. Practically, the sensors would need to be attached via more suitable mechanism that would prevent the dog chewing, tampering or destroying the attachment method when left unsupervised. On a processing level, further thought would need to be made on how to segment and compare measurements gathered while cornering to those gathered while walking straight. Movement at speed and the effects of fatigue upon gait would also be of interest for the deployment of this system in the measurement of gait in a more naturalistic setting than that used here [52,53,54]. Furthermore, an automatic mechanism for classifying gaits is needed such that inter-gait based measurements are not grouped. Once addressed the system can begin to be used to investigate gait abnormalities.

Throughout the evaluation experiments, much was learnt about the practicality of making such measurements; particularly the importance of the habituation period and the ability to accelerate it by encouraging movement and providing additional, engaging and distracting stimuli, such as toys and treats. The sensors used in the experiments featured wireless data capture. While convenient there was the occasion where the wireless range of the devices became prohibitive and thus on a practical level, it would be desirable to have a memory buffer storage option in future iterations. As previously stated the attachment method was also not ideal and will require further refinement prior to future deployments.

Although the system worked without bias on the healthy test dogs (which included a range of sizes and breeds), there is still significant work that needs to be done before the system is fit for purpose in a clinic. For example, as in human gait analysis, standardized collection protocols should be developed and fully benchmarked against. Such an exercise is the first step to being able to ascertain what a “normal” gait is on an inter-dog basis. Additionally, such standardization could assist in the investigation of the random variables leading to poor performance in a small portion of the sample. This should be accompanied by the collection of more detailed information regarding the dog’s physiology and history in an attempt to identify the cause of the errors reported here to more extensively correct, account for and recognize such problems in future deployments.

## 5. Conclusions

This research evaluated the base component of an IMU-based gait measurement system compatible for use with canines. These results demonstrate good sensitivity and repeatability at a precision that is likely to be sufficient to identify clinically relevant gait abnormalities in dogs. In human movement science, such systems have found uses in diagnostics, rehabilitation and research into intervention effectiveness. It is anticipated similar benefits could be realized if the evaluated detection methods were to be developed into an end-to-end solution for canine veterinary practice.

Once refined such a system will provide an exciting analogue to those systems currently showing extensive success in human research and medicine and will assist in providing further insight into the health and welfare of canines for use by their owners, veterinarians and researchers. Furthermore, the establishment of an open source solution for use with dogs could be adapted for use with other domestic quadruped mammals.

## Figures and Tables

**Table 1:** The mean error (seconds) for the initial and final contacts during both the walking and trotting gaits of each leg.

Foot	Walking FC	Walking IC	Trotting FC	Trotting IC
FL	0.042	0.015	0.018	0.028
FR	0.035	0.008	0.004	0.011
HL	0.084	−0.006	0.072	0.008
HR	0.071	0.012	0.056	0.012

## Appendix A

**Table A1:** Ethogram.

Annotated Gait Phases and Types	Description
Final Contact (FC)	The moment where all areas of the foot have left the ground. Signifies the start of the swing phase.
Initial Contact (IC)	The moment where the first area of the foot again contacts the ground. Signifies the end of the swing phase.
Swing Phase	The period of time between FC and IC where the focal foot is not in contact with the ground.
Walk	Gait pattern in which one leg is in the swing phase at any time and the other three support the body.
Trot	Gait pattern in which legs move in diagonal pairs (Front Left and Hind Right; Front Right and Hind Left).
